# Highly multiplexed immune profiling throughout adulthood reveals kinetics of lymphocyte infiltration in the aging mouse prostate

**DOI:** 10.18632/aging.204708

**Published:** 2023-05-13

**Authors:** Jonathan J. Fox, Takao Hashimoto, Héctor I. Navarro, Alejandro J. Garcia, Benjamin L. Shou, Andrew S. Goldstein

**Affiliations:** 1 Department of Molecular, Cell, and Developmental Biology, University of California, Los Angeles, CA 90095, USA; 2 Molecular Biology Interdepartmental Program, University of California, Los Angeles, CA 90095, USA; 3 Division of Hematology-Oncology, Department of Medicine, David Geffen School of Medicine, University of California, Los Angeles, CA 90095, USA; 4 Department of Urology, David Geffen School of Medicine, University of California, Los Angeles, CA 90095, USA; 5 Eli and Edythe Broad Center of Regenerative Medicine and Stem Cell Research, University of California, Los Angeles, CA 90095, USA; 6 Jonsson Comprehensive Cancer Center, University of California, Los Angeles, CA 90095, USA; 7 Molecular Biology Institute, University of California, Los Angeles, CA 90095, USA; 8 Current Address: Division of Biology and Biological Engineering, California Institute of Technology, Pasadena, CA 91125, USA; 9 Current Address: Keck School of Medicine, University of Southern California, Los Angeles, CA 90095, USA; 10 Current Address: Division of Cardiac Surgery, Department of Surgery, Johns Hopkins University School of Medicine, Baltimore, MD 21205, USA

**Keywords:** prostate, immune microenvironment, mass cytometry, inflammation, lymphocyte

## Abstract

Aging is a significant risk factor for disease in several tissues, including the prostate. Defining the kinetics of age-related changes in these tissues is critical for identifying regulators of aging and evaluating interventions to slow the aging process and reduce disease risk. An altered immune microenvironment is characteristic of prostatic aging in mice, but whether features of aging in the prostate emerge predominantly in old age or earlier in adulthood has not previously been established. Using highly multiplexed immune profiling and time-course analysis, we tracked the abundance of 29 immune cell clusters in the aging mouse prostate. Early in adulthood, myeloid cells comprise the vast majority of immune cells in the 3-month-old mouse prostate. Between 6 and 12 months of age, there is a profound shift towards a T and B lymphocyte-dominant mouse prostate immune microenvironment. Comparing the prostate to other urogenital tissues, we found similar features of age-related inflammation in the mouse bladder but not the kidney. In summary, our study offers new insight into the kinetics of prostatic inflammaging and the window when interventions to slow down age-related changes may be most effective.

## INTRODUCTION

Aging is associated with the dysfunction of many different cellular and molecular processes which results in the development of age-related pathologies including cancer [1]. Prostate cancer is the most common non-dermatologic cancer in men, and the risk of prostate cancer increases with age [2]; however, the mechanisms driving increased disease risk with age are poorly understood. One well-established aspect of aging which may contribute to disease risk is the development of chronic low-grade inflammation, termed “inflammaging” [3]. It is understood that chronic inflammation can promote cancer through a number of mechanisms including the secretion of pro-oncogenic cytokines, production of reactive oxygen and nitrogen species, induction of pro-tumorigenic cells, and immunosuppression of the anti-tumor response [4]. Indeed, it has been well established that inflammation is associated with focal areas of atrophic hyperplasia in the prostate which are enriched for potential tumor progenitors [5, 6], and mouse models of bacterial prostatitis have shown that inflammation can promote prostate cancer initiation [7, 8]. Recent work interrogating human prostate tissues with active bacterial infections identified ERG^+^ precancerous lesions, suggesting that bacterial infections can initiate gene fusions associated with prostate cancer [9]. Importantly, a 2014 clinical trial found that chronic inflammation in the benign prostate increases the risk for developing high-grade prostate cancer later in life, providing evidence for a link between prostatic inflammation and disease risk [10]. Our group has also shown that prostatic inflammation is associated with luminal progenitor cells that can serve as target cells for human prostate cancer initiation [11]. In both mice and humans, this population of luminal progenitor cells in the prostate increases with age, expanding the pool of potential target cells for neoplastic transformation [12].

While mice do not naturally develop prostate cancer, hormonal and genetic alterations in the prostate epithelium can cooperate with aging to promote tumorigenesis in the mouse prostate [13]. Importantly, the cellular changes that define aging phenotypes in the human prostate are conserved in the aging mouse prostate, indicating that the mouse is an ideal model system to investigate factors that regulate age-related changes to the prostate [12]. Like in humans, the aging mouse prostate is characterized by increased inflammation and stromal disorganization [14]. Compared to 3-month-old mice at the beginning of adulthood, the prostates of mice aged to 24 months are enriched for T and B lymphocytes [12]. Whether features of prostatic inflammation emerge primarily in old age or steadily increase throughout adulthood has not been established.

Efforts to slow age-related changes have garnered considerable interest over the past decade. Caloric restriction and mTOR inhibition show promise in delaying aging phenotypes and extending lifespan in model organisms [15, 16]. Pharmacological enhancement of NAD^+^ levels has also been shown to reverse aging phenotypes [17]. Importantly, anti-inflammatory strategies represent another option for combating the effects of aging [18]. Understanding the dynamics of age-related changes in model organisms is essential for defining the window in which interventions are most efficacious.

Recently, single-cell sequencing has been used to create *Tabula Muris Senis*, a valuable database of gene expression across 23 tissues in the aging mouse [19, 20]. Notably, the prostate was not one of the tissues profiled in this database. Additionally, the expression of mRNA is not always correlated with protein abundance [21]. Therefore, proteomic characterization of tissues, though often covering fewer genes than transcriptomic methods, provides useful information from which to infer cellular phenotype and function.

In this study, we sought to define the kinetics of mouse prostatic inflammation throughout adulthood at single-cell resolution using mass cytometry, or cytometry by time-of-flight (CyTOF), a proteomic method which has been previously used by our group and others to study immune cells in the prostate [12, 22, 23]. We also set out to determine how aging-associated inflammation in the prostate compares with other urogenital tissues susceptible to age-related cancers. Our study reveals that features of old age in the mouse prostate emerge far earlier than previously reported, with evidence of increased lymphocyte infiltration emerging between 6 and 12 months of age. Age-related changes in immune profiles were shared by the mouse prostate and bladder, suggesting common features of inflammaging in distinct genitourinary tissues.

## RESULTS

### Identification of distinct immune subsets in mouse prostate using mass cytometry

Prostates from mice of various ages between 1 and 16 months were collected and weighed to confirm the relationship between age and prostate development. We observed a positive correlation between age and wet prostate weight (ρ = 0.6673, *P*_ρ_ < 0.001) with a rapid increase in weight in pre-pubescent mice (<3 months old) (Supplementary Figure 1A), consistent with the rapid, androgen-dependent growth of the prostate which occurs postnatally until sexual maturity at 2–3 months of age [24, 25]. After reaching adulthood, the mouse prostate continues to grow at a much slower rate. In post-pubertal adult mice aged 3 to 16 months, we observed a positive correlation between age and prostate weight (ρ = 0.5144, *P*_ρ_ < 0.01), with a significant increase in mass at 16 months of age (*p* < 0.05) (Supplementary Figure 1A). Importantly, age was also positively correlated with the percentage of CD45^+^ immune cells in the prostate as measured by flow cytometry (ρ = 0.7034, *P*_ρ_ < 0.01) (Supplementary Figure 1B), consistent with age-related prostatic inflammation. The increase in total immune cells in the aged prostate was confirmed by quantifying the total lymphoid area in aged mouse prostates using immunohistochemistry (Supplementary Figure 1C, 1D).

**Figure 1:**
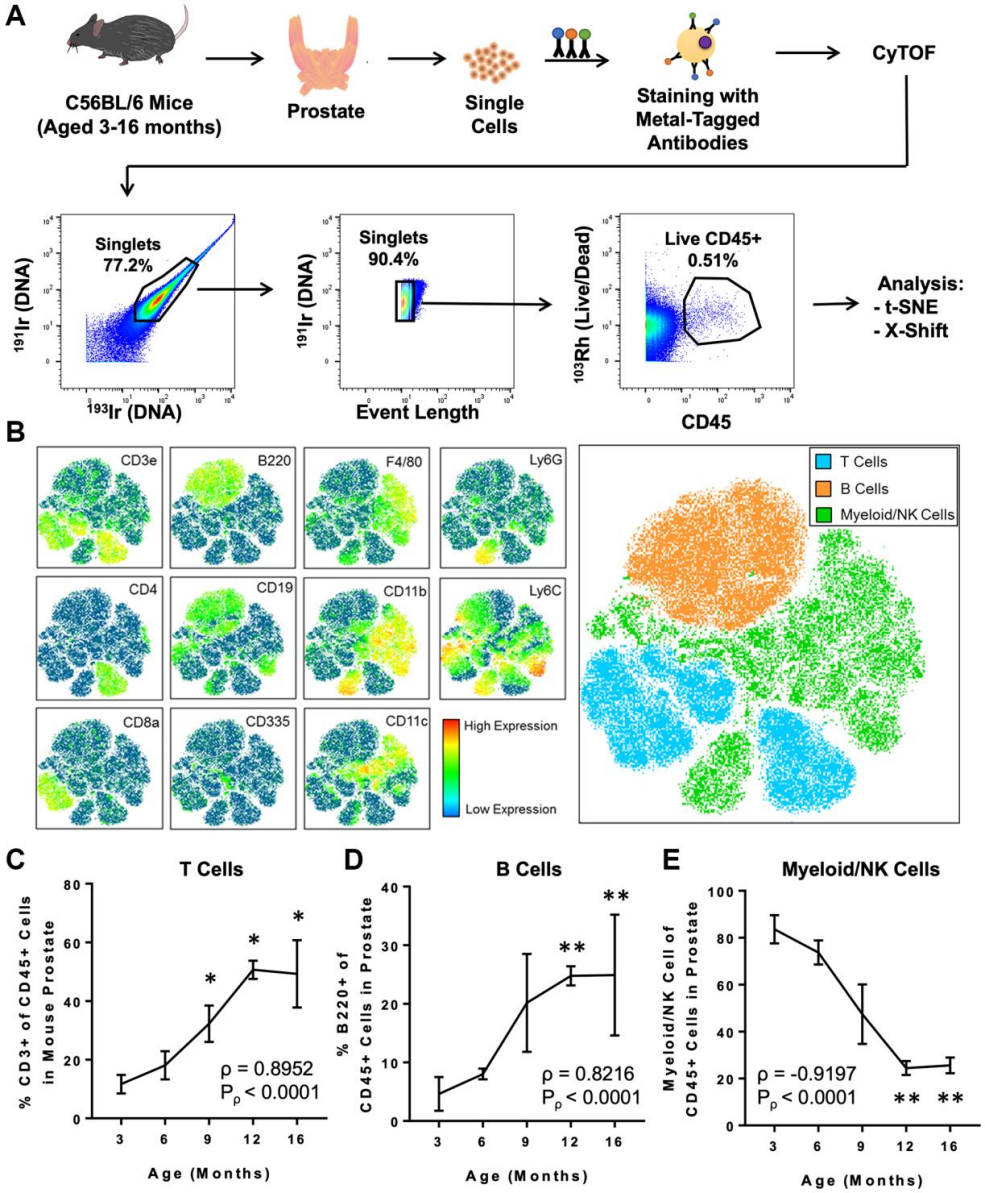
**CyTOF immunophenotyping of the aging mouse prostate.** (**A**) Workflow for prostate immunophenotyping using CyTOF. Mouse prostates of different ages were isolated, dissociated to single cells, and stained with a panel of metal-tagged antibodies before data acquisition via mass cytometry. Bivariate plots show gating for single, live CD45^+^ immune cells before analysis using clustering algorithms (t-SNE and X-Shift). Percentages represent the fraction of events that are within the gate in each bivariate plot. ^191^Ir/^193^Ir labels DNA of all cells to distinguish singlets from doublets. ^103^Rh labels DNA of dead cells. This flowchart was adapted from a previous publication from our group [12]. (**B**) t-SNE plot generated from the immune cells from mouse prostate, bladder, and kidney. Left: Heat maps showing expression of selected lineage markers by immune cells clustered using t-SNE. See Supplementary Figure 2A for the full set of markers. Right: t-SNE plot separated into 3 broad groups of immune cells (T cells, B cells, and myeloid/NK cells). (**C**–**E**) Quantification of changes to the mouse prostate immune cell composition during adult aging for CD3^+^ T cells (**C**), B220^+^ B cells (**D**), and myeloid/NK cells (**E**). Spearman correlation coefficient (ρ) and associated *p*-value (*P*_ρ_) represent the correlation between % immune cell type and age. Data represents mean ± SD of 4 biological replicates at each age. Kruskal-Wallis, *p* < 0.01 (T cells, B cells, and Myeloid/NK cells). Dunn’s multiple comparisons test against 3-months-old, ^*^p < 0.05, ^**^p < 0.01.

Next, we used CyTOF to characterize age-related changes in the immune microenvironment of the prostate throughout adulthood. Prostates were isolated from adult mice aged 3, 6, 9, 12, and 16 months (each *n* = 4), and dissociated cells were stained with a panel of 19 metal-tagged antibodies designed to label a broad variety of immune cell types using CyTOF (Table 1 and Figure 1A). Immune cells from all ages were clustered using the t-distributed stochastic neighbor embedding (t-SNE) algorithm for dimensional reduction and visualization (Figure 1A, 1B and Supplementary Figure 2A). Based on expression of well-defined immune cell lineage markers, the t-SNE plot was separated into 3 broad groups: T cells (expressing CD3e), B cells (expressing B220), and myeloid/NK cells (expressing CD335, F4/80, CD11b, CD11c, Ly6C, or Ly6G) (Figure 1B). We observed a positive correlation between age and the percentage of T cells (ρ = 0.8952, *P*_ρ_ < 0.0001) and B cells (ρ = 0.8216, *P*_ρ_ < 0.0001) in the adult mouse prostate (Figure 1C, 1D). The enrichment for T and B lymphocytes with age was accompanied by a respective decrease in the percentage of myeloid/NK cells (ρ = –0.9197, *P*_ρ_ < 0.0001) in the aging mouse prostate (Figure 1E). These trends are consistent with the age-related increase in T and B lymphocytes and decrease in myeloid and NK cells previously reported in 3- and 24-month-old mouse prostates by our group [12].

**Table 1:** CyTOF immunophenotyping antibody panel for discovery experiment.

**Label**	**Target**	**Clone**	**Conjugation**	**Source**
^89^Y	CD45	30-F11	Pre-conjugated	Fluidigm
^139^La	CD27	LG.3A10	Maxpar kit	BioLegend
^141^Pr	Ly6G	1A8	Pre-conjugated	Fluidigm
^146^Nd	F4/80	BM8	Pre-conjugated	Fluidigm
^147^Sm	CD80 (B7-1)	16-10A1	Maxpar kit	BioLegend
^148^Nd	CD11b	M1/70	Pre-conjugated	Fluidigm
^150^Nd	Ly6C	HK1.4	Pre-conjugated	Fluidigm
^152^Sm	CD3e	145-2C11	Pre-conjugated	Fluidigm
^153^Eu	CD274 (PD-L1)	10F.9G2	Pre-conjugated	Fluidigm
^154^Sm	CD152 (CTLA-4)	UC10-4B9	Pre-conjugated	Fluidigm
^155^Gd	CD25	3C7	Maxpar kit	BioLegend
^156^Gd	CD4	RM4-5	Maxpar kit	BioLegend
^159^Tb	CD279 (PD-1)	29F.1A12	Maxpar kit	BioLegend
^166^Er	CD19	6D5	Pre-conjugated	Fluidigm
^167^Er	CD335 (NKp46)	29A1.4	Pre-conjugated	Fluidigm
^168^Er	CD8a	53-6.7	Pre-conjugated	Fluidigm
^172^Yb	CD86 (B7-2)	GL1	Pre-conjugated	Fluidigm
^176^Yb	CD45R (B220)	RA3-6B2	Pre-conjugated	Fluidigm
^209^Bi	CD11c	N418	Pre-conjugated	Fluidigm

**Figure 2:**
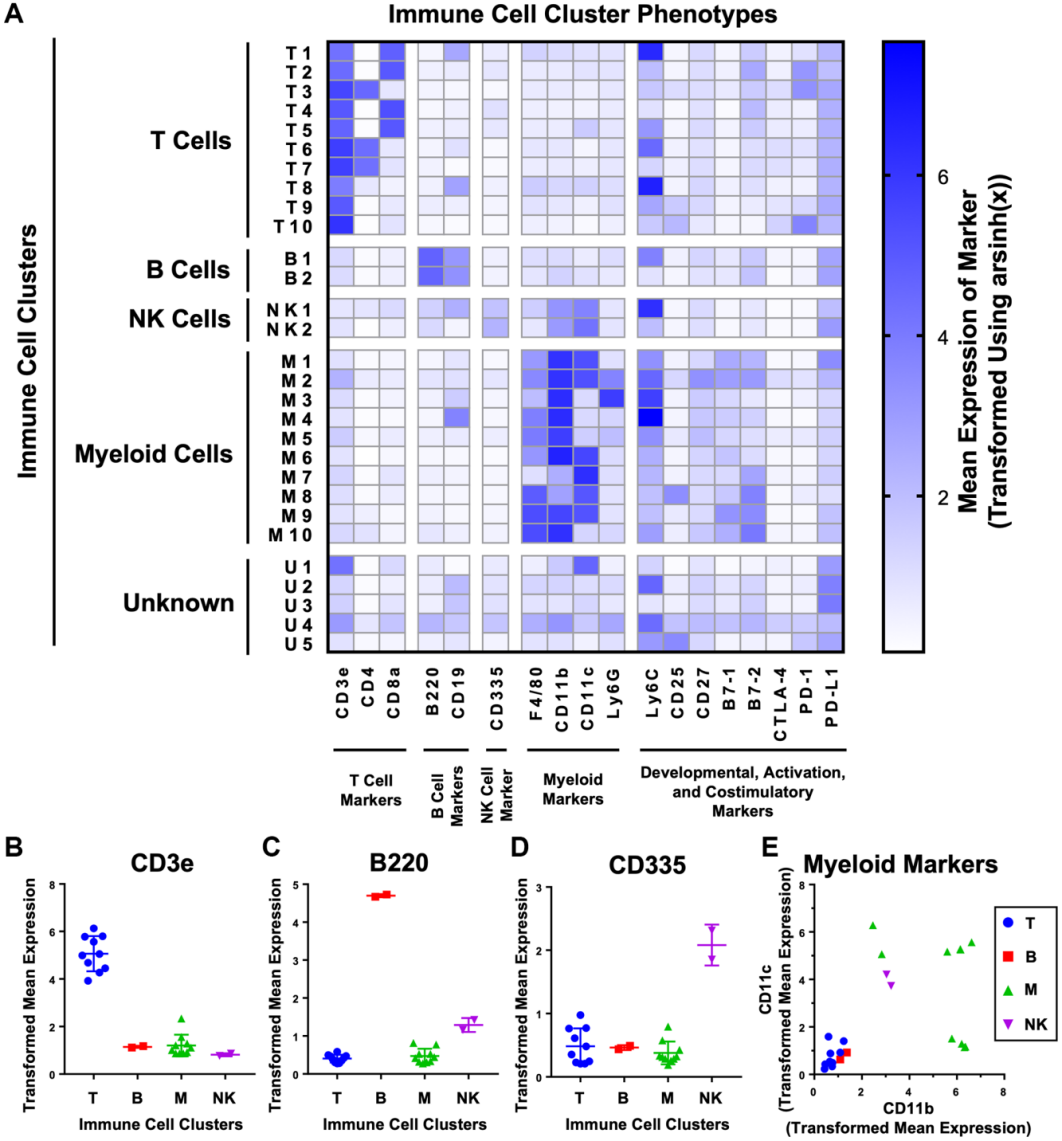
**Unsupervised clustering of mouse urogenital immune cells identifies 29 phenotypically distinct clusters.** (**A**) Heat map showing the phenotypes of the 29 clusters generated on immune cells detected from aging mouse prostates, bladders, and kidneys using CyTOF. Clusters were separated into T cells (CD3, CD4, CD8), B cells (B220, CD19), NK cells (CD335), myeloid cells (F4/80, CD11b, CD11c, and Ly6G), and unknown by expression of immune lineage markers. Shading represents mean marker expression transformed by arsinh(x). (**B**–**E**) Immune cell cluster expression of lineage markers for T cells (CD3) (**B**), B cells (B220) (**C**), NK cells (CD335) (**D**), and myeloid cells (CD11b and CD11c) (**E**). Data represents mean marker expression in each cluster transformed by arsinh(x) ± SD between clusters of the same broad immune cell type. Kruskal-Wallis, *p* < 0.001 (CD3), *p* < 0.05 (B220), *p* = 0.106 (CD335). Two-way ANOVA, *p* < 0.0001 (myeloid markers).

To provide more granularity to our analysis we performed unsupervised k-nearest neighbors clustering using the X-Shift algorithm to further subdivide the t-SNE plot into phenotypically-distinct immune cell clusters (Figure 2A and Supplementary Figure 2B). The X-Shift algorithm was chosen because it optimizes the numbers of clusters to prevent over- and under-clustering [26]. We identified 29 distinct immune cell clusters which were then subdivided by cell type. Expression of lineage markers was used to identify 24 of 29 immune cell clusters as T cells (CD3e^+^), B cells (B220^+^ and CD19^+^), NK cells (CD335^+^), and myeloid cells (any combination of F4/80^+^, CD11b^+^, CD11c^+^, or Ly6G^+^) (Figure 2A–2E). The 5 remaining clusters were classified as unknown due to expression of both T cell and myeloid cell markers (U1), lack of a strong signal for any lineage-specific markers (U2–U3, U5) or mild, nonspecific staining for every marker (U4) (Figure 2A).

Surprisingly, there were 6 immune cell clusters with staining for both Ly6C and CD19 (T1, T8, B1, NK1, M4, U2) (Figure 2A). Since CD19 is only expected on B cells, expression by other immune cell lineages is unlikely [27]. Instead, we determined that there was interference between the Ly6C and CD19 channels in our experiment. At high levels of Ly6C, there was overflow of signal into the CD19 channel, resulting in a linearly proportional increase in signal between the two channels (Supplementary Figure 3A). In our panel, Ly6C was conjugated to neodymium-150, and CD19 was conjugated to erbium-166 (Table 1). These two lanthanide isotopes differ by 16 Da, consistent with known effects of oxidation of neodymium-150 by oxygen-16 as the sample is ionized to plasma in the mass cytometer (Supplementary Figure 3B) [28]. We checked every other combination of the 19 markers used and found no other instances of signal spillover (Supplementary Figure 3C). The effects of signal spillover from Ly6C into CD19 taken into account, high Ly6C expression likely caused the T1, T8, NK1, M4, and U2 clusters to appear as CD19^+^. However, expression of the B cell specific marker B220 by cluster B1 indicates that its CD19 signal is likely true [29]. Interestingly, Ly6C is expressed across many different immune lineages but is not expressed on resting B cells [30, 31]; however, Ly6C has been found in some cases to be upregulated in B cells exposed to lipopolysaccharide and type III interferon [32, 33]. Given that CD19 is not expressed in immune cells besides B cells, the artifactual signal interference from Ly6C into CD19 is not likely to have affected the clustering or cell type identification in our analysis.

### Aging-related changes of mouse prostatic immune cell populations arise early in adulthood

**Figure 3:**
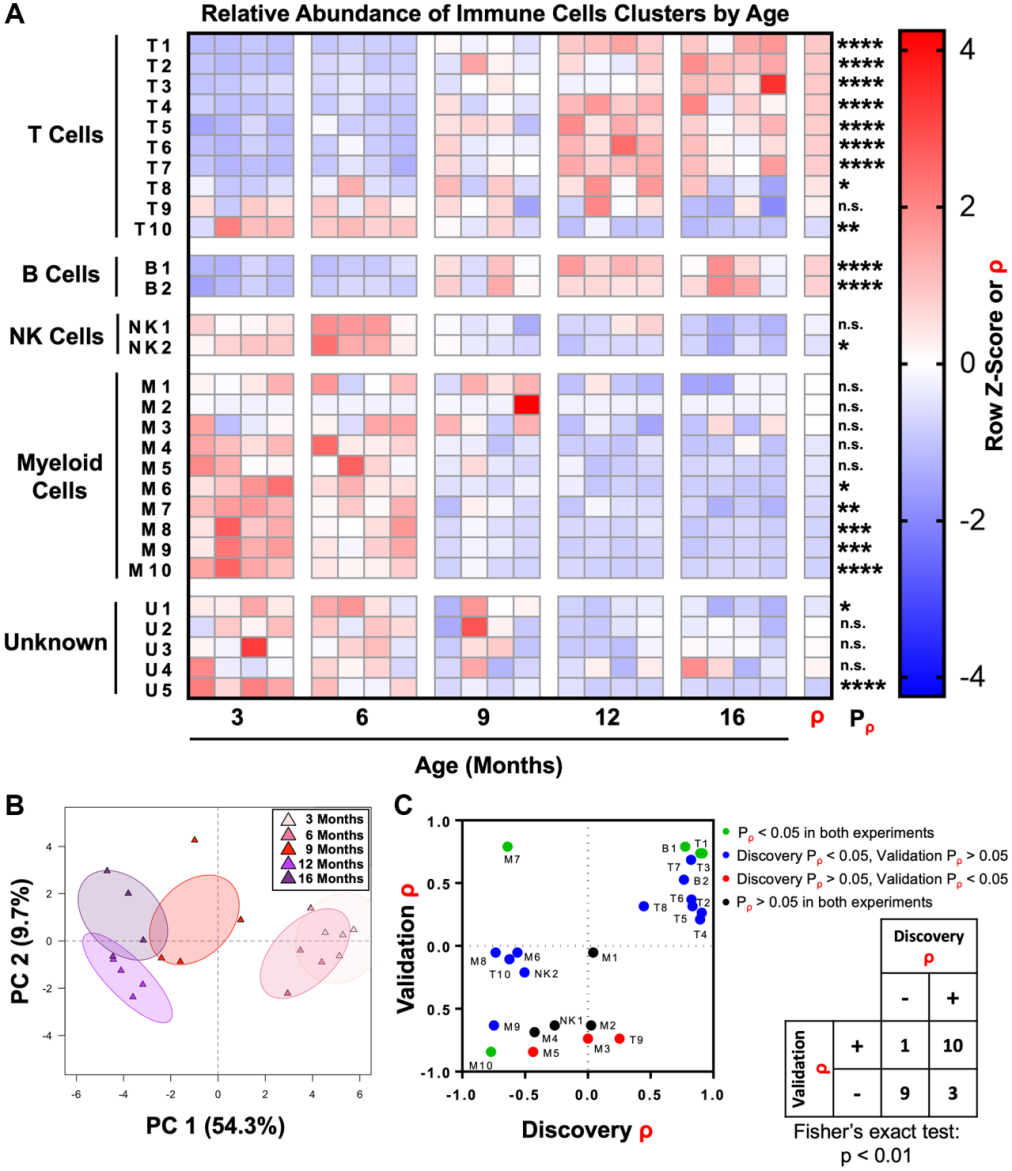
**The adult mouse prostate immune microenvironment changes progressively with age.** (**A**) Heat map showing changes to immune cell cluster abundance in the aging adult mouse prostate, correlation with age (ρ), and associated *p*-value (*P*_ρ_). Shading indicates abundance represented as a row z-score except where Spearman correlation (ρ) is indicated. Data represents 4 biological replicates at each age. ^*^p < 0.05, ^**^p < 0.01, ^***^p < 0.001, ^****^p < 0.0001. n.s., not significant, *p* ≥ 0.05. (**B**) Principal component analysis (PCA) was performed on immune cell cluster frequencies for mouse prostates at different ages. Ellipses represent 95% confidence intervals for 4 biological replicates at each age. Abbreviation: PC: principal component. (**C**) Twenty-four clusters of immune cells were identified in a separate validation experiment with prostates from mice 4-, 9-, and 15-months-old (each *n* = 3). These clusters were matched to clusters in the discovery experiment, and correlation with age (ρ) and associated *p*-value (*P_ρ_*) were calculated. Left: Dot plot compares correlation coefficients for each immune cell cluster in the initial (discovery) and validation experiments. Coloring of points represents significance (*P_ρ_*). Right: Contingency table comparing the direction of correlation with age (ρ > 0 or ρ < 0) irrespective of *p*-value for immune cell clusters between the discovery and validation experiments. Cluster M3 was not counted because ρ = 0 in the discovery experiment. Fisher’s exact test was performed to evaluate the distribution of clusters in the four quadrants of the graph/contingency table, *p* < 0.01.

Having used unsupervised clustering to subdivide immune cells in the mouse prostate into distinct subsets, we next examined how these populations change throughout adulthood. Correlation analysis was performed to determine whether each immune cell cluster was enriched (ρ > 0) or depleted (ρ < 0) with age. We found that 19/29 immune cell clusters were significantly correlated with age (ρ ≠ 0, *P*_ρ_ < 0.05). Consistent with the overall increase in T and B lymphocytes in the mouse prostate with age, 8/10 T cell clusters and 2/2 B cell clusters were significantly enriched with age. Similarly, we found that 1/2 NK cell clusters and 5/10 myeloid cell clusters had a significant negative correlation with age (Figure 3A and Supplementary Table 1). Excluding an extreme outlier in the abundance of cluster M2 in one of the 9-month-old mice, we also found a significant negative correlation (*P*_ρ_ < 0.05) between M2 abundance in the prostate and age (Supplementary Figure 4).

Principal component analysis was performed to assess the progression of changes in the mouse prostate immune microenvironment. Prostates from 3- and 6-month-old mice clustered together as did those from 12- and 16-month-old mice, demonstrating the similarity in immune microenvironments of prostates in the youngest and oldest age groups (Figure 3B). Prostates from 9-month-old mice clustered between the other ages, demonstrating an intermediate immune phenotype at this age. These results show that the shift in the inflammatory microenvironment of the aging mouse prostate occurs between 6 and 12 months of age.

To validate these changes to the immune microenvironment of the mouse prostate, we performed a separate CyTOF immunophenotyping experiment with a panel of the same markers and mice 4-, 9-, and 15-months-old (each *n* = 3) (Supplementary Table 2). The 24 known immune cell clusters from the discovery experiment were matched to immune cells from the validation experiment, and Spearman correlation analysis was performed (Supplementary Figure 5 and Supplementary Table 3). Four of the 24 immune cell clusters (T1, T3, B1, and M10) were validated (same sign for ρ and *P*_ρ_ < 0.05 in both discovery and validation experiments) (Figure 3C). Fifteen of the 24 immune cell clusters had the same direction of correlation with age in both the discovery and validation experiments but failed to meet the barrier of statistical significance in both experiments. The majority of these were significant in the discovery but not the validation experiments, likely due to the low statistical power of *n* = 3 in the validation. Overall, there was a statistically significant correlation between the sign of ρ for immune cell clusters in each experiment (*p* < 0.01, Fisher’s exact test), supporting the reproducibility of our CyTOF immunophenotyping (Figure 3C).

**Figure 4:**
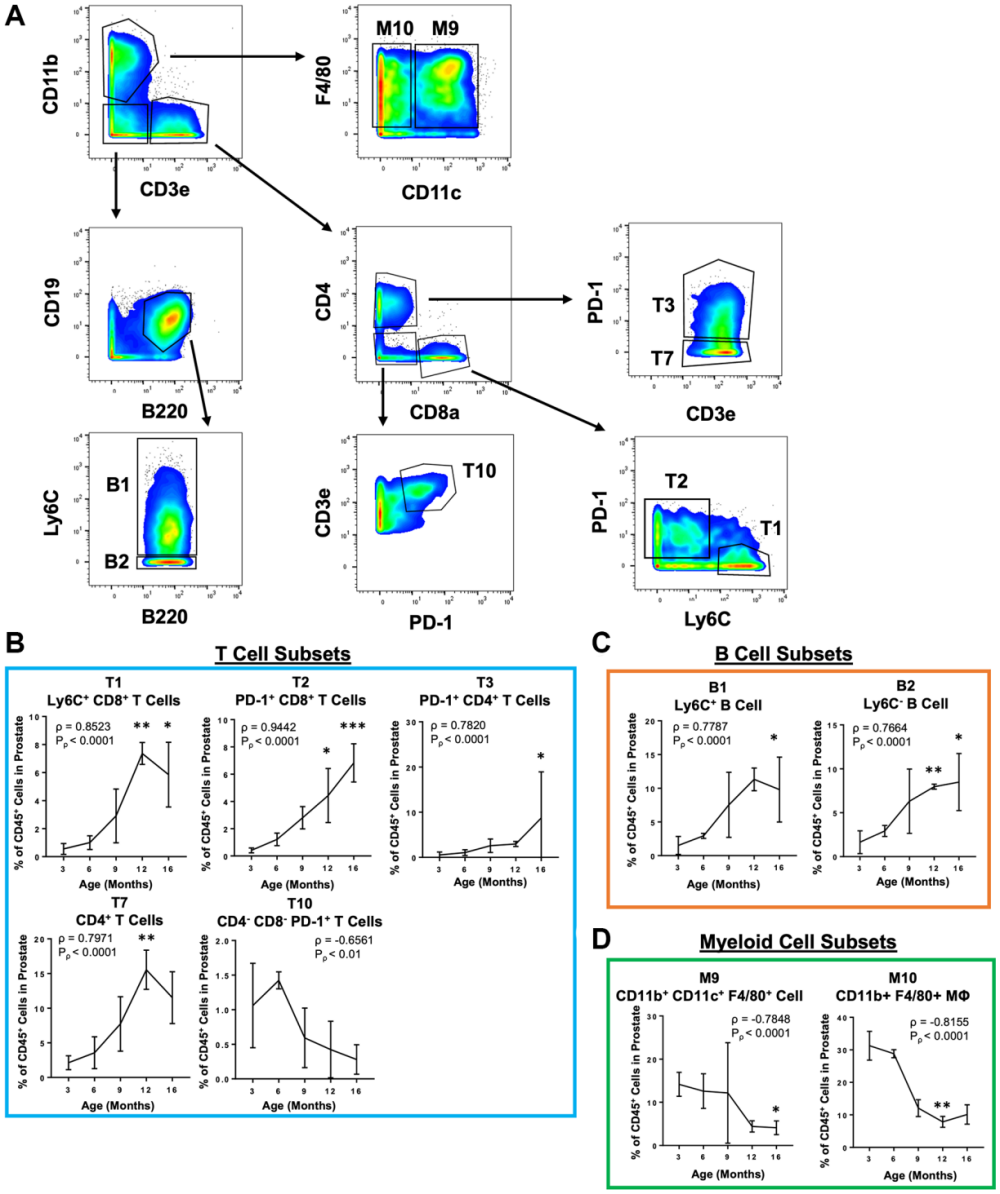
**Simplified gating scheme to identify immune cell clusters that change in abundance with age in the mouse prostate.** (**A**) Bivariate plots showing a gating scheme using a simplified 11 marker panel (CD45, CD3e, CD4, CD8a, CD19, B220, F4/80, CD11b, CD11c, Ly6C and PD-1) to identify 4 validated immune cell clusters (T1, T3, B1, and M10) and 5 others (T2, T7, T10, B2, and M9) in the validation CyTOF experiment. (**B**–**D**) Quantification of immune cell subset abundance by proportion of total CD45^+^ cells in the mouse prostate identified using the simplified 11 marker panel separated into T cells (**B**), B cells (**C**), and myeloid cells (**D**). Data represents mean ± SD of 4 biological replicates at each age. MΦ, macrophage. Spearman correlation coefficient (ρ) and associated *p*-value (*P*_ρ_) represent the correlation with age. Kruskal-Wallis, *p* < 0.05 (T3, T10, B1, B2, M9), *p* < 0.01 (T1, T2, T7, M10). Dunn’s multiple comparisons test against 3-months-old, ^*^p < 0.05, ^**^p < 0.01, ^***^p < 0.001.

Because CyTOF is not a widely available technique, we sought to provide a more accessible way to study these age-related immune microenvironment changes in the mouse prostate. Guided by the phenotypes of the validated immune cell clusters, a simpler gating scheme was constructed which can identify the 4 validated clusters (T1, T3, B1, and M10) and 5 additional clusters that were significant in the discovery experiment (T2, T7, T10, B2, and M9) using a reduced number of markers (Table 2 and Figure 4A). The reduced panel has fewer markers to be used with more widely accessible 12-color flow cytometry [34]. Importantly, manual gating to reidentify these immune cell clusters in the discovery experiment produced populations with significant correlations with age that are consistent with correlations found using X-Shift clustering (Figure 4B–4D).

**Table 2:** Simplified panel for identifying immune cells in the mouse prostate that change with age.

**Marker**
CD45
CD3e
CD4
CD8a
F4/80
CD11b
CD11c
B220
CD19
Ly6C
PD-1

### Mouse prostate and bladder share features of inflammaging

**Figure 5:**
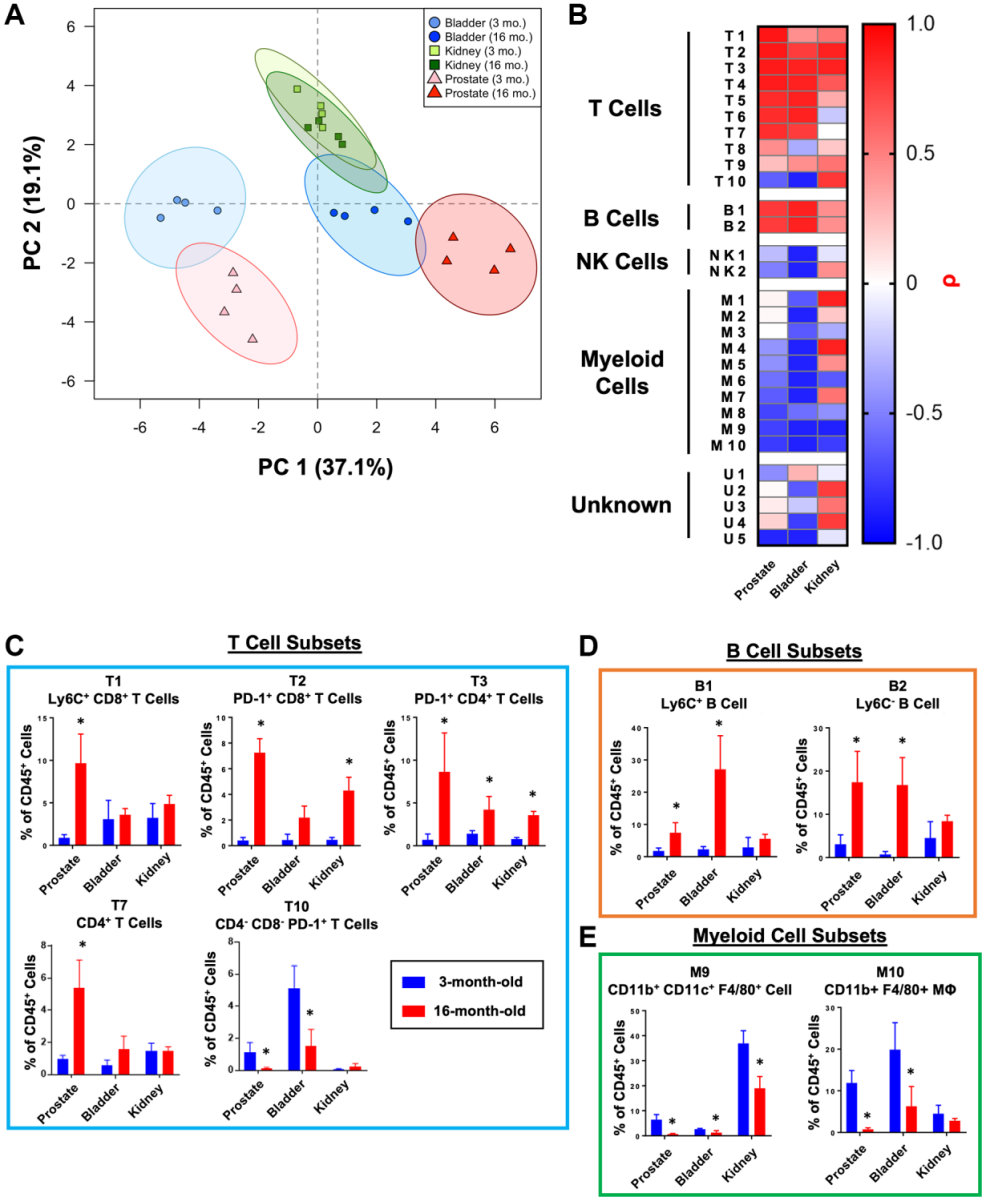
**Comparison of age-related changes to the immune microenvironment of the mouse bladder, kidneys, and prostate.** (**A**) Principal component analysis (PCA) was performed on immune cell cluster frequencies for mouse prostates, bladders, and kidneys at 3- and 16-months-old. Ellipses represent 95% confidence intervals for 4 biological replicates at each age and tissue. Abbreviation: PC: principal component. (**B**) Heat map showing correlations with age (ρ) for immune cell clusters from mouse prostate, bladder, and kidneys irrespective of *p*-value. Shading represents Spearman correlation (ρ). (**C**–**E**) Quantification of immune cell cluster abundance at 3 and 16 months of age in mouse prostate, bladder, and kidney separated into T cells (**C**), B cells (**D**), and myeloid cells (**E**). Mann Whitney *U* test between 3- and 16-months-old, ^*^p < 0.05. Data represents mean ± SD of 4 biological replicates at each age and tissue.

As part of the discovery experiment, we also performed CyTOF on bladders and kidneys from the same 3- and 16-month-old mice (each *n* = 4) to compare aging of the prostate to other urogenital tissues with age-related cancer risk [35, 36]. Immune cells from these tissues were included for X-Shift clustering so that we could examine age-related changes to the same 29 immune cell clusters as the prostate (Supplementary Table 1). In the mouse bladder, we identified significant correlations between age and 17/29 immune cell clusters (ρ ≠ 0, *P*_ρ_ < 0.05) (Supplementary Figure 6A). Unlike the prostate and bladder, the immune profiles of the 3- and 16-month-old mouse kidneys displayed more similarity, with only 5/29 immune cell clusters significantly correlated with age (Supplementary Figure 6B). Principal component analysis was performed on the 3- and 16-month-old samples from the prostate, bladder, and kidney to assess the similarities in how different urogenital tissues age (Figure 5A). Three-month-old bladder and prostate clustered together as did 16-month-old bladder and prostate, suggesting these tissues are similar in how their immune microenvironments change with age. On the other hand, both ages of kidney clustered together near the 16-month-old bladder, suggesting that the kidney immune microenvironment changes less with age and was most similar to the 16-month-old bladder.

To better understand these relationships, we compared the correlation coefficients (ρ) for each immune cell cluster, irrespective of *p*-value, in the prostate, bladder, and kidney (Figure 5B). Thirteen of 29 clusters were correlated with age in the same direction in all three tissues (all ρ > 0 or ρ < 0) (T1–T4, T9, B1–B2, NK1, M6, M8–M10, and U5). Among the other immune cell clusters, prostate and bladder shared more similarities to each other than to kidney (Figure 5B–5E and Supplementary Figure 6C). These results demonstrate that different mouse urogenital tissues have distinct inflammatory signatures that change with age and that the aging prostate more closely resembles the aging bladder than the kidney.

## DISCUSSION

Inflammation in the prostate and aging are both associated with an increased risk for developing prostate cancer [10, 37]. Our group and others have previously reported that the mouse prostate experiences an increase in immune cells as it ages, however the dynamics of how these changes occur were unclear [12, 14]. In this study, we characterized how the inflammatory microenvironment of the adult mouse prostate changes during aging using highly-multiplexed single-cell mass cytometry. This dataset presents the most comprehensive profiling of the aging adult mouse prostate immune profile to date.

By 3 months of age, male mice are sexually mature and the prostate has completed development [38]. At this age, myeloid cells make up the vast majority of immune cells in the mouse prostate (Figure 1E). Throughout adulthood, the mouse prostate undergoes enrichment for T and B lymphocytes, resulting in a profound shift in the tissue’s immunological milieu (Figure 1C, 1D). Interestingly, this age-related expansion of lymphocytes in the prostate happens in contrast to the age-related myeloid bias of hematopoiesis [39]. As mice age, different processes including bone marrow stem cell niche remodeling, inflammatory cytokines, and telomere dysfunction skew hematopoiesis towards myeloid lineages [40–42]. Aging is also associated with involution of the thymus, resulting in reduced production of naïve T cells [43]. Additionally, B cell development is impaired in aged mouse bone marrow [44]. These many processes converge to reduce lymphocyte output and increase myeloid differentiation in the aging mouse. In contrast, the enrichment for lymphocytes in the aging mouse prostate suggests that the prostate contains a unique aging signature compared to other tissues.

We used 19 surface markers to identify 29 phenotypically-distinct clusters of immune cells in the mouse prostate (Figure 2). We then tracked how the abundance of each immune cell cluster changes during adult mouse aging by comparing tissue aged 3-, 6-, 9-, 12-, and 16-months. We determined that there is a shift in the immune microenvironment of the mouse prostate which takes place between 6 and 12 months of age (Figure 3A, 3B). A separate validation experiment demonstrated that the majority of age-related changes to specific immune cell clusters were reproducible (Figure 3C). One possible mechanism driving this increased inflammation is the enhanced CD4^+^ T helper 17 cell response in aged mice which can contribute to an increased recruitment of lymphocytes and other immune cells [45, 46]. While lymphocytic infiltrates are also characteristic of viral infections, this explanation is unlikely given the reproducibility of this shift across multiple experiments and separate groups of researchers. Nonetheless, as a gland of the urogenital track, the prostate can be exposed to and infected by various viruses. Intriguingly, there have been multiple studies showing that a subset of prostate cancers in humans is associated with infection by human papilloma virus and a number of *Herpesviridae* [47–49]. Future research may benefit from the development of mouse models of chronic viral infection in the prostate, which may shed more light on the drivers of altered immune microenvironment in older mouse prostates.

While there is an overall enrichment for T and B lymphocytes in the aging mouse prostate, analysis of these changes at the more granular level of immune cell cluster shows greater complexity in these changes to the aging immune microenvironment. Though all B cell clusters were enriched with age, we identified T cell clusters which did not change or were reduced in abundance with age. Similarly, while the majority of NK and myeloid cell clusters decreased in proportion with age, we found NK and myeloid cell clusters which did not significantly change in proportion with age (Figure 3A). These findings clarify that a subset of immune cells clusters accounts for the overall increase in T and B lymphocytes and decrease in NK and myeloid cells with age. Notably, age is correlated with an enrichment for CD4^+^ and CD8^+^ T cells expressing the inhibitory checkpoint receptor programmed cell death protein 1 (PD-1) in the mouse prostate (Figure 4B). PD-1 is upregulated in activated T cells, and it plays a central role in regulating the T cell immune response [50, 51]. PD-1 is also known as a marker for “exhausted” T cells in the context of chronic inflammation [52]. Previous studies using mouse splenocytes and peripheral blood have also shown aging to be associated with increased PD-1 expression on CD4^+^ and CD8^+^ T cells [53, 54]. Indeed, PD-1^+^ CD4^+^ and CD8^+^ T cells were enriched in all three urogenital tissues analyzed (Figure 5C), indicating that this PD-1^+^ T cell aging signature is likely not specific to the prostate. Nevertheless, our demonstration of increased PD-1^+^ T cells in the aging mouse prostate may still be useful information given the recent interest in developing immunotherapies targeting the PD-1/PD-L1 axis in prostate cancer [55–57].

In this study we tracked how immune cell populations changed with age using whole mouse prostate gland. Notably, there are distinct morphological differences between the mouse and human prostate gland. While the human prostate is a single gland with distinct zones, the mouse prostate is instead comprised of four lobes with different functions. Genetically and physiologically, the dorsal and lateral lobes of the mouse prostate most closely correspond to the peripheral zone of the human prostate where the majority of prostate adenocarcinomas arise [58, 59]. While we used whole mouse prostate to more comprehensively map changes to the aging prostate immune microenvironment, the relevance of this data to the field of human prostate inflammation is limited by these morphological differences between species. Characterizing age related immune changes in each lobe of the mouse prostate would be useful in determining whether the changes reported in this study are specific to a particular lobe or is a feature of the gland as a whole. Another limitation of this study was that CyTOF requires dissociation of the tissue to a single-cell suspension using mechanical and enzymatic means. As a result, we were only able to profile immune cells that could survive the dissociation protocol. Between different organs and ages, we assume that different immune cell types are equally likely to survive the dissociation protocol, allowing for relative comparisons to still be made. Other multiplexed approaches that preserve tissue architecture, like imaging mass cytometry (IMC) or sequential fluorescence *in situ* hybridization (seqFISH), would be necessary to detect cell types that may not survive long enough for CyTOF detection.

Though we used highly-multiplexed CyTOF to study these changes to the prostate immune microenvironment, we sought to devise a simplified panel that could be accessible with more widely available technologies. We took the most reproducible immune cell cluster changes and designed a panel of 11 markers which can reidentify the same immune cell clusters that are significantly correlated with age (Table 2 and Figure 4). This reduced panel could be used to study changes to the mouse prostate immune microenvironment using more accessible 12-color flow cytometry [34].

Finally, we used CyTOF to survey the immune microenvironment of the aging mouse bladder and kidney to determine how the aging phenotype of the prostate compares to other urogenital tissues. We found that the prostate aging immune signature is more similar to the bladder than the kidney (Figure 5 and Supplementary Figure 6). The proximity of the prostate and bladder may explain their similar aging immune profiles. Additionally, both tissues are derived from the urogenital sinus, whereas the kidney develops from the ureteric bud and metanephric mesenchyme [60, 61]. The similarity in aging immune signature between mouse prostate and bladder may also be reflective of their shared developmental origin. Understanding what drives similar age-related changes in the prostate and bladder may reveal common approaches to prevent inflammaging and reduce age-related disease risk.

## MATERIALS AND METHODS

### Animal work

Male C57BL/6J and C57BL/6N mice from Jackson Laboratories and the UCLA Department of Radiation Oncology’s animal core facility were used in this study. The UCLA Division of Laboratory Animal Medicine (DLAM) bred and maintained the mice. The UCLA Animal Research Committee (ARC) and DLAM approved all animal protocols.

### Tissue isolation and dissociation to single cells

Male mice of different ages were euthanized using carbon dioxide asphyxiation, then their urogenital tract and right kidneys were removed. Under a microscope, microdissection was performed to isolate the prostate and bladder from the urogenital tract. Tissues were mechanically dissociated with a razor blade then enzymatically dissociated in RPMI 1640 (Gibco) containing 10% fetal bovine serum (FBS), 1 mg/mL collagenase type I (Gibco), and 0.1 mg/mL DNase (Sigma) at 37°C on a nutating platform for 60–90 minutes. After washing the cell pellets with 1X DPBS (Gibco), cells were resuspended in 37°C TrypLE Express Enzyme, no phenol red (Thermo Fisher Scientific) for 5 minutes before being quenched by adding RPMI 1640. Cells were further disrupted using the shear stress generated by drawing through an 18G syringe. Finally, cells were sequentially passed through 100 μm and 70 μm cell strainers (Corning) to produce a single-cell suspension.

### Flow cytometry

Single-cell suspensions of 5 × 10^4^–1 × 10^6^ cells from mouse prostate tissue of different ages (each *n* = 4) were stained in cell staining buffer comprised of 1X DPBS (Gibco) with 5 g/L protease-free bovine serum albumin (Sigma-Aldrich) and 200 mg/L sodium azide (Sigma-Aldrich). Nonspecific antibody binding by Fc receptors was blocked using TruStain fcX (anti-mouse CD16/32) Antibody (BioLegend) according to the manufacturer’s protocol. Cells were then stained with rat anti-CD49f-PE (BioLegend), rat anti-CD326 (EpCAM)-APC/Cy7 (BioLegend), goat anti-Trop2-APC (R&D Systems), and rat anti-CD45-FITC (BioLegend) for 30 minutes at room temperature. Cells were washed with cell staining buffer then fixed in 1% paraformaldehyde (Electron Microscopy Sciences) for 10–15 minutes at 37°C. After fixation, cells were chilled on ice for 1 minute, washed with cell staining buffer, and stored at 4°C before analysis on a FACSCanto flow cytometer (BD Biosciences).

### Immunohistochemistry

Immunohistochemistry was performed as described previously [62] with some modifications. Paraffin sections of the mouse prostate were deparaffinized and underwent epitope unmasking using a heat antigen retrieval step. Section-staining was performed using the manufacturer’s protocol for Mouse and Rabbit Specific HRP/DAB IHC Detection Kit - Micro-polymer (Abcam ab236466). Antibodies used were: rabbit anti-CD45 (Abcam ab208022, 1:1000), anti-CD3 (Abcam ab5690, 1:300), and anti-CD19 (Cell Signaling 90176, 1:800). Hematoxylin and eosin (H&E) staining was performed by UCLA’s Translational Pathology Core Laboratories. Entire areas of an H&E-stained prostate section were captured by Axio Imager M2 (Zeiss) from two separate sections from a prostate, from four animals per age group. The sectional areas of the tertiary lymphoid structures was calculated using Zen 3.5 software (Zeiss).

### Antibodies for mass cytometry

Antibody panels were designed using the Maxpar Panel Designer software (Fluidigm) to check for interference between the channels. The discovery and validation experiments used the same list of markers but differed in the metal tags conjugated to the antibodies (Table 1 and Supplementary Table 2). Antibodies were purchased pre-conjugated from Fluidigm or conjugated at the UCLA Jonsson Comprehensive Cancer Center (JCCC) and Center for AIDS Research Flow Cytometry Core Facility using the Maxpar X8 Polymer Chemistry kit (Fluidigm) following the manufacturer’s instructions. Antibody cocktails were prepared by adding 1.1 μL/sample of the conjugated antibody stock to a single tube then diluting to a volume of 50 μL/sample with cell staining buffer comprised of 1X DPBS (Gibco) with 5 g/L protease-free bovine serum albumin (Sigma-Aldrich) and 200 mg/L sodium azide (Sigma-Aldrich).

### Cell surface staining for mass cytometry

Single-cell suspensions of 3 × 10^5^ – 1.8 × 10^6^ cells from mouse prostate, bladder, and kidney tissue of different ages were stained in cell staining buffer comprised of 1X DPBS (Gibco) with 5 g/L protease-free bovine serum albumin (Sigma-Aldrich) and 200 mg/L sodium azide (Sigma-Aldrich). Staining was performed in a 96-well V-bottom plate. The discovery experiment was performed using prostates from 3-, 6-, 9-, 12-, and 16-month-old mice; bladders from 3- and 16-month-old mice; and kidneys from 3- and 16-month-old mice (each *n* = 4). The validation experiment was performed using prostates from 4-, 9-, and 15-month-old mice (each *n* = 3). To distinguish between live and dead cells, cells were washed with cell staining buffer, resuspended in 200 μL cell staining buffer containing 1 μM Cell-ID Intercalator-103Rh (Fluidigm), and incubated for 15 minutes at 37°C. Staining with rhodium-103 was quenched with 2 mL cell staining buffer. Next, cells were centrifuged at 400 × g for 5 minutes, resuspended in 50 μL cell staining buffer containing 1 μg/mL TruStain fcX (anti-mouse CD16/32) Antibody (BioLegend), and incubated at room temperature for 10 minutes. Next, 50 μL of the diluted antibody cocktail was added to each sample, and cells were incubated at room temperature for 30 minutes. Cells were washed twice with cell staining buffer then resuspended in 200 μL cell intercalation solution comprised of 1X DPBS containing 1.6% paraformaldehyde and 125 nM Cell-ID Intercalator-Ir (Fluidigm). Samples were incubated at 4°C for 12–48 hours then washed sequentially with cell staining buffer and 1X DPBS. To remove clumps of cells, samples were passed through a 40 μm cell strainer (Corning) before a final wash with MilliQ water (Millipore). After the final wash, cells were resuspended in 50 μL MilliQ water for analysis via mass cytometry.

### Mass cytometry

Mass cytometry was performed with a Helios mass cytometer (Fluidigm) at the UCLA Jonsson Comprehensive Cancer Center (JCCC) and Center for AIDS Research Flow Cytometry Core Facility. Samples were washed twice with Maxpar cell staining buffer (Fluidigm) and twice with MilliQ water (Millipore). Next, samples were resuspended in 10% EQ Four Element Calibration Beads (Fluidigm) containing natural abundance cerium (140Ce/142Ce), europium (151Eu/153Eu), holmium (165Ho), and lutetium (175Lu/176Lu). Samples were run at an event rate of 300–500 events/second, and data were normalized using bead-based normalization in the CyTOF software.

### Mass cytometry data analysis

Analysis of mass cytometry data was performed in FlowJo V10 (FlowJo LLC). Single, live CD45+ immune cells from each sample in the discovery and validation experiments were manually gated. Each sample was given a unique Sample ID for later deconvolution, then all CD45+ cells from samples in the discovery experiment were concatenated into a single .fcs file. The native FlowJo t-distributed stochastic neighbor embedding (t-SNE) function was used to perform dimensional reduction on the single file containing immune cells from all samples in discovery experiment, using all markers besides CD45 and the following settings: Iterations, 3000; Perplexity, 30; Eta (learning rate), 200. Heat maps of marker expression on the t-SNE plot were generated using the FlowJo Color Map Axis function. Unsupervised k-nearest neighbors (KNN) clustering was performed using the X-Shift algorithm plugin for FlowJo [26]. Plugins for FlowJo are found on the FlowJo Exchange and utilize R (R Core Team). X-Shift clustering was performed using all markers besides CD45 and the following settings: number nearest neighbors (K), 54; distance metric, angular; subsampling limit, 42,790; Run ID, auto. Cluster phenotypes were calculated by taking the geometric mean of marker expression by cells in each cluster then transforming using arsinh(x). Immune cell clusters were manually classified as T cells, B cells, NK cells, myeloid cells, and unknown based on expression of the known lineage markers CD3e, CD4, CD8, B220, CD19, CD335, F4/80, CD11b, CD11c, and Ly6G. Phenotypes of immune cell clusters in the discovery experiment were used to manually gate for matched immune cells in the validation experiment. Cluster abundance was calculated by determining the number of cells from each cluster in each sample using the unique Sample ID, then dividing by the total number of CD45+ immune cells in each sample. Age-related immune cell cluster enrichment was analyzed by calculating Z-scores for cluster abundance, using the mean and standard deviation of all replicates and ages for a given cluster. N x N plots were made in FlowJo using the Layout Editor function. Heat maps of cluster phenotype and enrichment were generated in Prism V7 (GraphPad).

### Principal component analysis

Principal component analysis (PCA) was performed using immune cell cluster abundance data from the discovery experiment. PCA was performed in R (version 3.6.2) using the singular value decomposition method on scaled matrix data.

### Statistical analysis

All statistical tests were performed in Prism V7 (GraphPad). Correlations with age were calculated using the non-parametric Spearman correlation coefficient (ρ) with a two-tailed test for significance (*P*_ρ_). The symbol “*P*_ρ_” was used instead of “*p*” to denote the *p*-value for correlation analysis due to the visual similarity to the symbol for the Spearman correlation coefficient “ρ”.

Comparisons between ages were made using the non- parametric Kruskal-Wallis H test followed by Dunn’s multiple comparisons test against the 3-month-old samples, using adjusted *p*-values to account for multiple comparisons. Kruskal-Wallis and two-way ANOVA were used to evaluate differences in immune lineage markers between immune cell clusters. Fisher’s exact test was used to compare the direction of correlation with age (ρ > 0 or ρ < 0) for immune cell clusters between the discovery and validation experiments. Mann Whitney *U* test was used to compare immune cell cluster frequencies at 3- and 16-months-old for each tissue. Number of replicates (n) and type of replicate are listed in the figure legends. Error bars represent SD. ^*^p < 0.05, ^**^p < 0.01, ^***^p < 0.001, ^****^p < 0.0001. n.s., not significant, *p* ≥ 0.05.

## Supplementary Materials

Supplementary Figures

Supplementary Table 1

Supplementary Table 2

Supplementary Table 3
