# Supplementary material for: Highly multiplexed immune profiling throughout adulthood reveals kinetics of lymphocyte infiltration in the aging mouse prostate
Source: Aging (Albany NY). 2023 May 13;15(9):3356–80. doi: 10.18632/aging.204708 (PMC10449296; doi:10.18632/aging.204708)
Supplement: Supplementary Figures [file aging-15-204708-s001.pdf]

SUPPLEMENTARY FIGURES

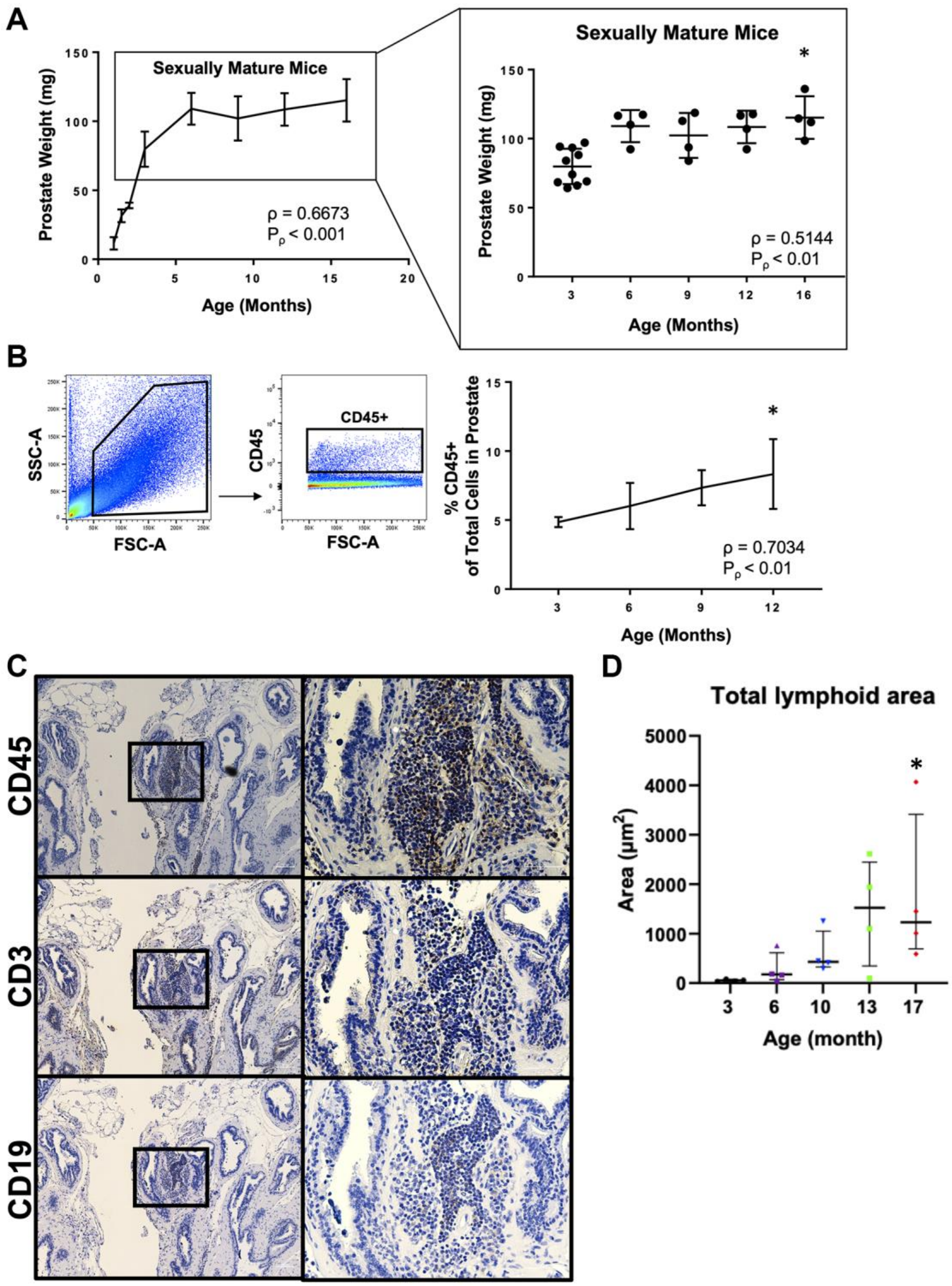

Supplementary Figure 1. Increased weight and inflammation in the aging adult mouse prostate. (**A**) Wet weight of prostates from mice of different ages. Left: Prostate weights of mice aged 1–16 months. Right: Prostate weights of sexually mature mice aged 3–16 months. Data represents mean ± SD of 2–10 biological replicates at each age. Spearman correlation coefficient (ρ) and associated *p*-value (P_ρ) represent the correlation with age. Kruskal-Wallis, $p < 0.05$ (sexually mature mice). Dunn's multiple comparisons test against 3-month-old mice, $^*p < 0.05$. (**B**) Analysis of immune cells in the prostate by flow cytometry. Left: Gating scheme to identify immune cells ($CD45^+$). Abbreviations: FSC-A: forward scatter area; SSC-A: side scatter area. Right: Quantification of $CD45^+$ immune cell frequency in the mouse prostate. Data represents mean ± SD of 4 biological replicates at each age. Spearman correlation coefficient (ρ) and associated *p*-value (P_ρ) represent the correlation with age. Kruskal-Wallis, $p < 0.05$. Dunn's multiple comparisons test against 3-month-old mice, $^*p < 0.05$. (**C**) Immunohistochemistry (IHC) of 15-month-old mouse prostate with staining for CD45 (pan-immune), CD3 (T cells), and CD19 (B cells). Each

marker is on a separate row, with images on the right magnified regions highlighted on the left. Scale bar, 100 μm for full images, 50 μm for magnified images. (**D**) Quantification of total lymphocyte area (μm^2) by IHC in prostates of mice aged 3, 6, 10, 13, and 17 months. Data represents mean ± SD of 4 biological replicates at each age. Dunn's multiple comparisons test against 3-month-old mice, $^{*}p < 0.05$.

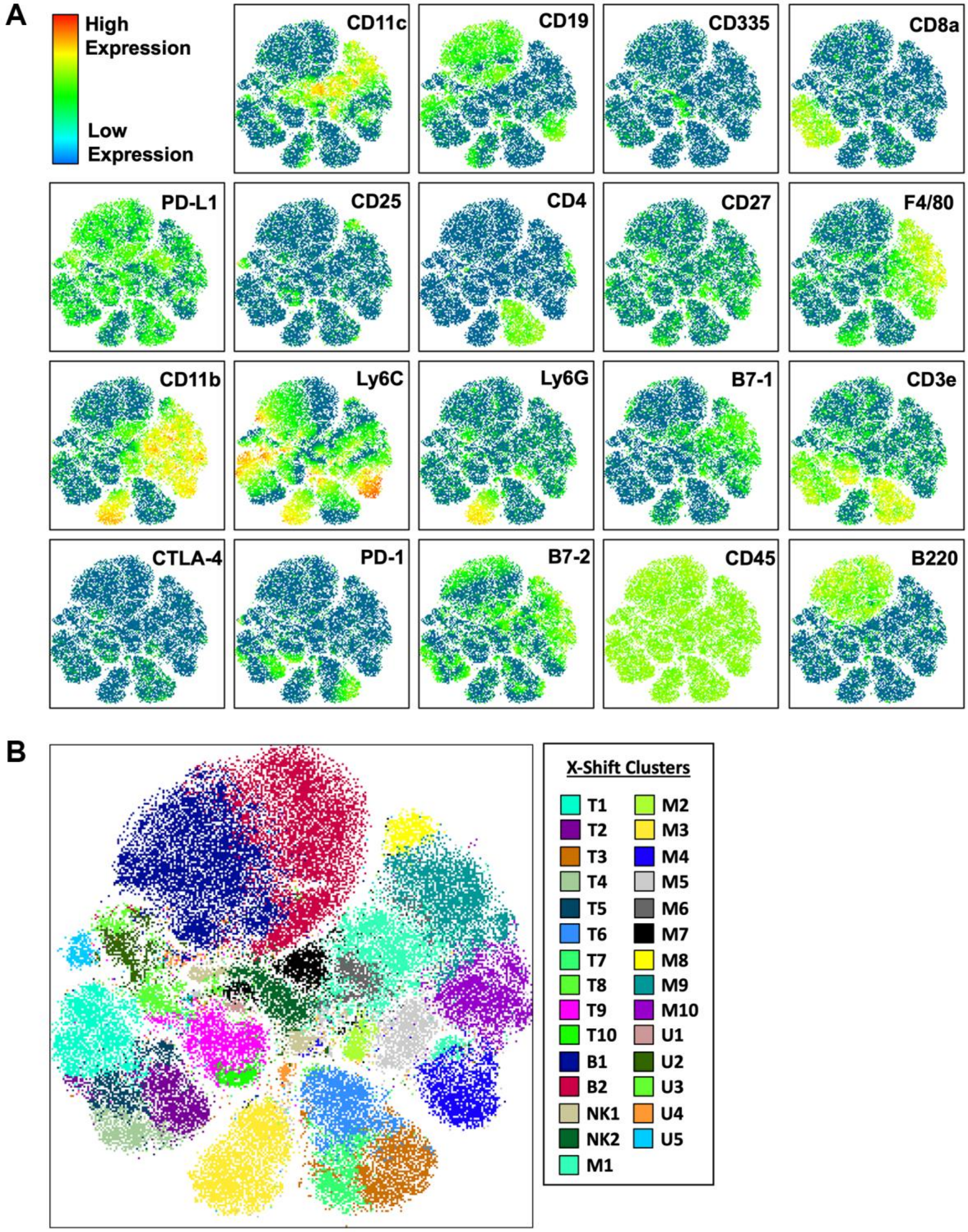

Supplementary Figure 2. Visualizing the heterogeneity of immune cells in mouse urogenital tissues with dimensional reduction and clustering algorithms. (**A**) t-SNE plot generated from the immune cells from mouse prostate, bladder, and kidney. Color mapping shows expression of each of the 19 markers in the CyTOF panel. (**B**) t-SNE plot showing 29 X-Shift generated immune cell clusters.

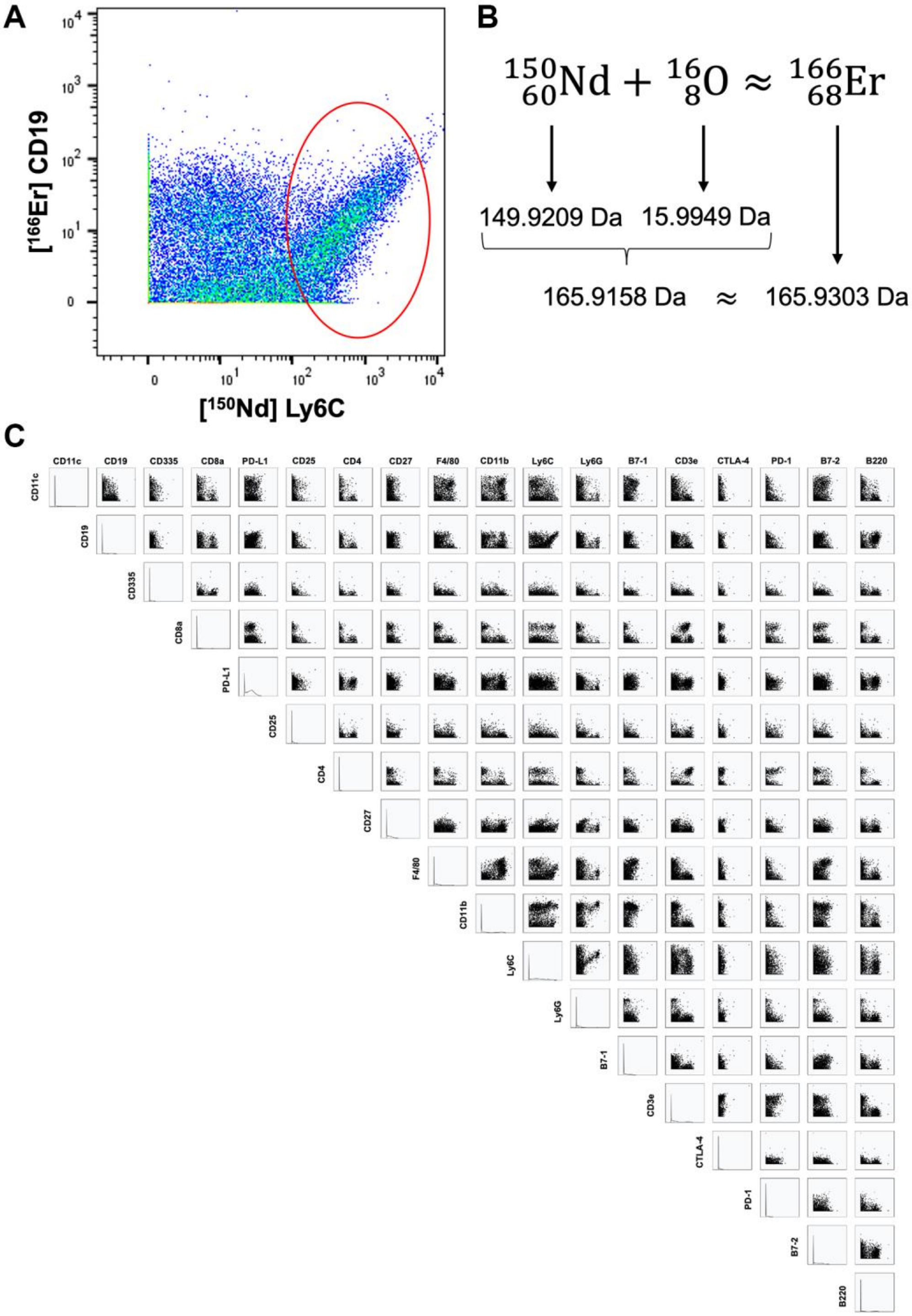

Supplementary Figure 3. Oxidation of the neodymium-150 ion leads to spillover into the CD19 channel. (**A**) Bivariate plot between [^{150}Nd] Ly6C and [^{166}Er] CD19 CyTOF channels showing all CD45^{+} cells from the discovery experiment. The red ellipse identifies the area on the plot in which there is a proportional increase in both channels starting only at high levels of [^{150}Nd] Ly6C signal. (**B**) When neodymium-150 is oxidized by oxygen-16, the most abundant isotope of oxygen, the resulting molecule contains 68 protons and 98 neutrons for a total mass of 166 Da. When detected by the time-of-flight mass spectrometer, the oxidized neodymium-150 has approximately the same apparent mass as erbium-166, causing spillover of the [^{150}Nd] Ly6C signal into [^{166}Er] CD19 when Ly6C is very highly expressed. Masses of isotopes were retrieved from PubChem [1]. (**C**) N x N plot showing all possible bivariate comparisons between the 19 CyTOF markers used in the discovery experiment. Boxes comparing a marker to itself show histograms of that marker's expression by immune cells in the discovery experiment.

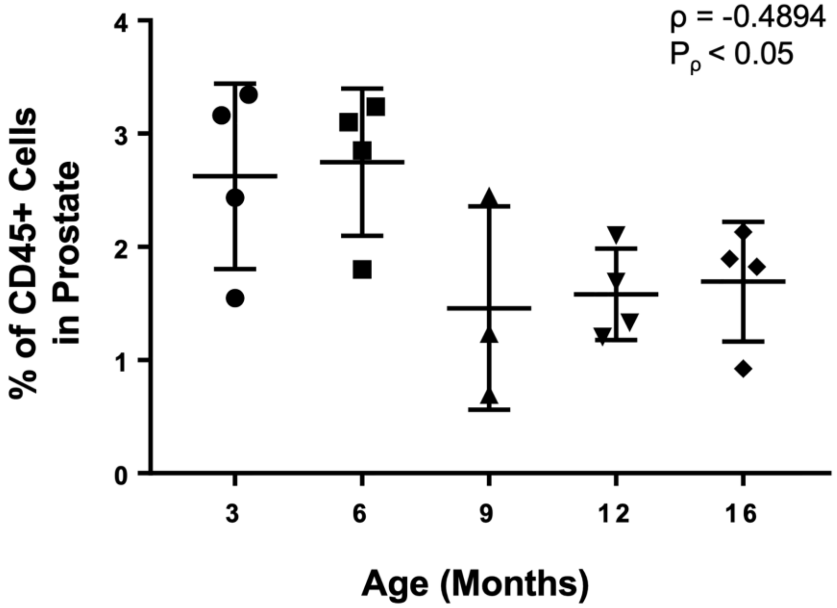

Supplementary Figure 4. Analysis of cluster M2 frequency in mouse prostate without an outlier. Quantification of % cluster M2 of $CD45^+$ cells in the aging mouse prostate without an outlier at 9-months-old. Spearman correlation coefficient (ρ) and associated *p*-value ($P_ρ$) represent the correlation with age. Data represents mean ± SD of 3–4 biological replicates at each age.

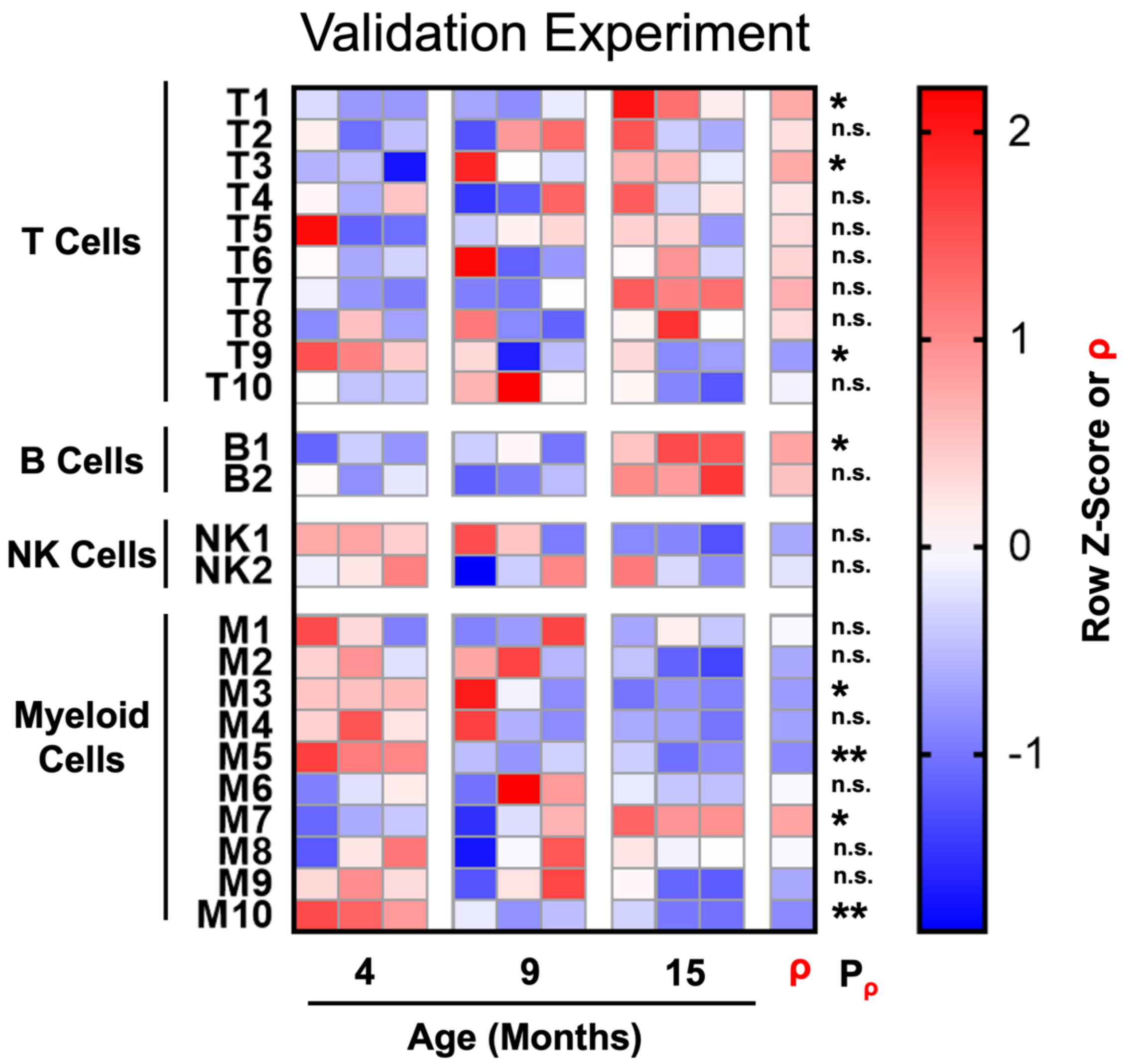

Supplementary Figure 5. Age-related immune cell cluster enrichment in the prostates of mice in the validation experiment. A separate validation experiment was performed, using CyTOF to profile immune cells in the prostates of mice ages 4-, 9-, and 15-months-old with a panel containing the same markers as the discovery experiment. Heat map shows changes to matched immune cell cluster abundance in the aging adult mouse prostate, correlation with age (ρ), and associated *p*-value (P_ρ). Shading indicates abundance represented as a row z-score except where Spearman correlation (ρ) is indicated. Data represents 3 biological replicates at each age. $^{*}p < 0.05$, $^{**}p < 0.01$. n.s., not significant, $p \geq 0.05$.

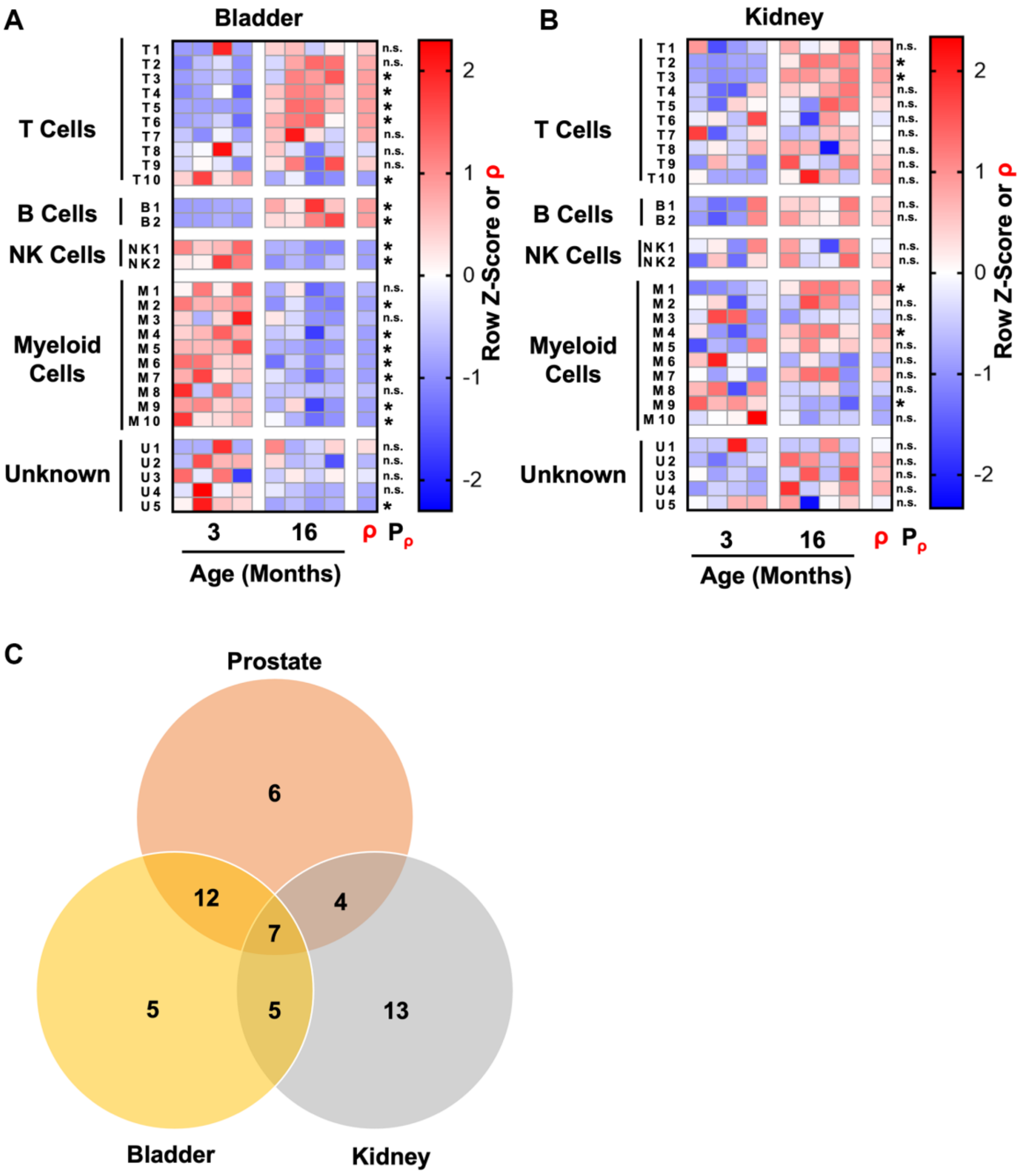

Supplementary Figure 6. Mouse prostate and bladder share more age-related changes to their immune microenvironment with each other than to kidney. (**A**, **B**) Heat maps showing changes to immune cell cluster abundance in the aging adult mouse bladder (**A**) and kidneys (**B**), correlation with age (ρ), and associated *p*-value (P_ρ). Shading indicates abundance represented as a row z-score except where Spearman correlation (ρ) is indicated. Data represents 4 biological replicates at each age. $^*p < 0.05$. n.s., not significant, $p \geq 0.05$. (**C**) Venn diagram showing the age-related immune cluster changes shared between the mouse prostate, bladder, and kidneys. An age-related immune cell cluster change is considered shared if each tissue has the same sign for the correlation with age (ρ both positive or negative) and if both are significant ($P_\rho < 0.05$). Clusters with no significant correlation with age in multiple tissues are also considered shared.

SUPPLEMENTARY REFERENCE

1. Kim S, Chen J, Cheng T, Gindulyte A, He J, He S, Li Q, Shoemaker BA, Thiessen PA, Yu B, Zaslavsky L, Zhang J, Bolton EE. PubChem 2019 update: improved access to chemical data. Nucleic Acids Res. 2019; 47:D1102–9.
https://doi.org/10.1093/nar/gky1033
PMID:30371825
