# Supplementary material for: Highly multiplexed immune profiling throughout adulthood reveals kinetics of lymphocyte infiltration in the aging mouse prostate
Source: Aging (Albany NY). 2023 May 13;15(9):3356–80. doi: 10.18632/aging.204708 (PMC10449296; doi:10.18632/aging.204708)
Supplement: Supplementary Table 2 [file aging-15-204708-s003.pdf]

Supplementary Table 2. CyTOF immunophenotyping antibody panel for validation experiment.

Label	Target	Clone	Conjugation	Source
^{89}Y	CD45	30-F11	Pre-conjugated	Fluidigm
^{139}La	CD27	LG.3A10	Maxpar kit	BioLegend
^{143}Nd	CD86 (B7-2)	GL1	Maxpar kit	BioLegend
^{146}Nd	F4/80	BM8	Pre-conjugated	Fluidigm
^{147}Sm	CD80 (B7-1)	16-10A1	Maxpar kit	BioLegend
^{148}Nd	CD11b	M1/70	Pre-conjugated	Fluidigm
^{150}Nd	Ly6C	HK1.4	Pre-conjugated	Fluidigm
^{151}Eu	Ly6G	1A8	Pre-conjugated	Fluidigm
^{152}Sm	CD3e	145-2C11	Pre-conjugated	Fluidigm
^{153}Eu	CD335 (NKp46)	29A1.4	Pre-conjugated	Fluidigm
^{154}Sm	CD152 (CTLA-4)	UC10-4B9	Pre-conjugated	Fluidigm
^{155}Gd	CD25	3C7	Maxpar kit	BioLegend
^{156}Gd	CD4	RM4-5	Maxpar kit	BioLegend
^{159}Tb	CD279 (PD-1)	29F.1A12	Maxpar kit	BioLegend
^{166}Er	CD19	6D5	Pre-conjugated	Fluidigm
^{168}Er	CD8a	53-6.7	Pre-conjugated	Fluidigm
^{169}Tm	CD274 (PD-L1)	10F.9G2	Maxpar kit	BioLegend
^{176}Yb	CD45R (B220)	RA3-6B2	Pre-conjugated	Fluidigm
^{209}Bi	CD11c	N418	Pre-conjugated	Fluidigm
